# Optomechanical microrheology of single adherent cancer cells

**DOI:** 10.1063/1.5010721

**Published:** 2018-03-05

**Authors:** Olaoluwa O. Adeniba, Elise A. Corbin, Randy H. Ewoldt, Rashid Bashir

**Affiliations:** 1 Department of Mechanical Science and Engineering, University of Illinois Urbana-Champaign, Urbana, Illinois 61801, USA; 2 Micro and Nanotechnology Laboratory, University of Illinois Urbana-Champaign, Urbana, Illinois 61801, USA; 3 Cardiovascular Institute, Perelman School of Medicine, University of Pennsylvania, Philadelphia, Pennsylvania 19104, USA; 4 Department of Mechanical Engineering and Applied Mechanics, School of Engineering and Applied Sciences, University of Pennsylvania, Philadelphia, Pennsylvania 19104, USA; 5 Department of Bioengineering, University of Illinois Urbana-Champaign, Urbana, Illinois 61801, USA; 6 Carle Illinois College of Medicine, University of Illinois Urbana-Champaign, Urbana, Illinois 61801, USA

## Abstract

There is a close relationship between the mechanical properties of cells and their physiological function. Non-invasive measurements of the physical properties of cells, especially of adherent cells, are challenging to perform. Through a non-contact optical interferometric technique, we measure and combine the phase, amplitude, and frequency of vibrating silicon pedestal micromechanical resonant sensors to quantify the “loss tangent” of individual adherent human colon cancer cells (HT-29). The loss tangent, a dimensionless ratio of viscoelastic energy loss and energy storage — a measure of the viscoelasticity of soft materials, obtained through an optical path length model, was found to be 1.88 ± 0.08 for live cells and 4.32 ± 0.13 for fixed cells, revealing significant changes (p < 0.001) in mechanical properties associated with estimated nanoscale cell membrane fluctuations of 3.86 ± 0.2 nm for live cells and 2.87 ± 0.1 nm for fixed cells. By combining these values with the corresponding two-degree-of-freedom Kelvin-Voigt model, we obtain the elastic stiffness and viscous loss associated with each individual cell rather than estimations from a population. The technique is unique as it decouples the heterogeneity of individual cells in our population and further refines the viscoelastic solution space.

## INTRODUCTION

The mechanical response of soft materials is generally reported as the frequency-dependent viscoelastic behavior determined using rheological techniques. However, performing rheology on living cells is particularly challenging due to invasiveness of applied forces and choosing appropriate time-dependent loading conditions to probe viscous material properties.^1^ Recently, it has been shown that the mechanical properties of cells are directly correlated with biological processes, such as in cancer^2–4^ and blood diseases.^5,6^ For example, normal cells are known to be stiffer than their cancerous counterparts. In practice, these mechanical biomarkers have the potential to contribute to early detection and diagnostic techniques.

In the past few years, there have been a variety of experimental techniques and models to measure and describe the rheological behavior of cells and tissue under different physiological conditions. These quantitative experimental techniques can be divided into two main categories: passive and active. Passive techniques involve the measurement of thermal fluctuations of embedded particles.^7^ Approaches for active microrheology generally involve the application of a local force and the measurement of material response. Some of these prominent techniques for active microrheology measurements include atomic force microscopy,^8^ cell poking,^9^ shear flow cytometry,^10^ microplates,^11–13^ optical tweezers,^14–16^ optical stretchers,^17,18^ magnetic tweezers,^19,20^ and optomechanical techniques.^21^ These techniques probe the behavior of cells at different length scales and timescales and employ different stress-strain magnitudes and behaviors.

In this paper, we present a technique for measuring the viscoelastic properties of individual adherent cells using a microresonator sensor. This technique simply combines the simultaneous measurement of the resonant frequency shift,^22^ amplitude change,^23^ and mechanically driven optical path phase^21^ within a cell using a microresonator to more uniquely determine the viscoelastic properties of a single cell without requiring averaging over a population of cells. This overcomes limitations of our previous techniques that depended on additional measurements of the average cell height, reference measurements, and other ensemble average cell group measurements.^23^ Here, we measure the loss tangent of adherent colon cancer (HT-29) cells, a descriptor of the relative viscoelastic properties of a cell. Using this technique, we are also able to quantify the nanoscale membrane fluctuations (height oscillations) of individual live HT-29 cells and their stiffer fixed counterparts.

## RESULTS

**FIG. 1.**
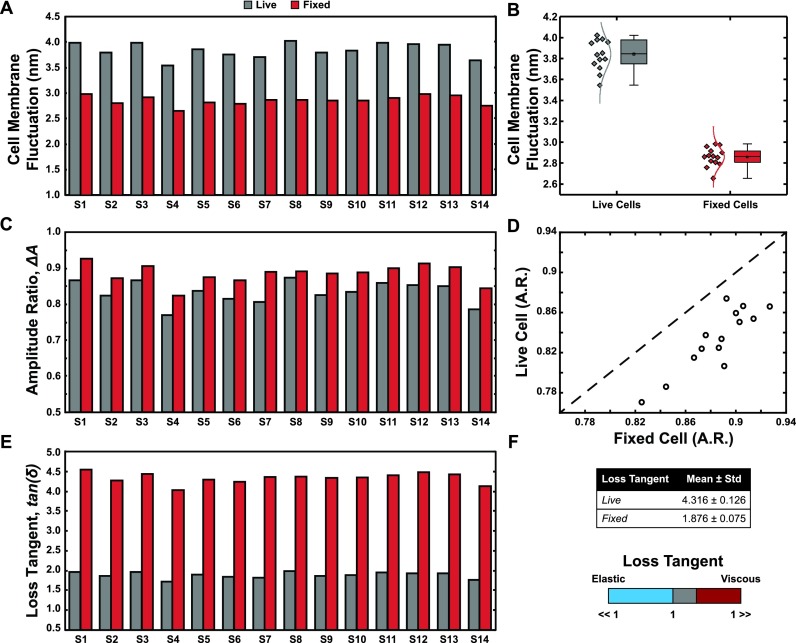
(a) Cell membrane fluctuations (height oscillation), *A_c_*, of 14 live and fixed cells validating 2DOF Kevin-Voigt simulation. (b) Boxplot providing a summary of the spread of membrane fluctuation, *A_c_*. (c) Experimental amplitude ratios for live and fixed cells. A higher amplitude ratio implies that a relative increase in the refractive index (and viscoelasticity) exceeds the relative change in the actual cell-sensor amplitude ratio. (d) The dotted line of unity slope comparing the plot of live amplitude against fixed amplitude ratios shows a significant difference between the ratio before and after fixation. (e) Estimated loss tangent, $\text{tan}(\delta )=\frac{c_{2}\omega }{k_{2}},$ a relative viscoelastic descriptor for live and fixed cells at their individual sensor resonant frequencies, ω. (f) Scale bar from Elastic to Viscous showing where both live and fixed cells fall on the range of values.

Figure 1(a) depicts an increase in the membrane fluctuation amplitude of the live cells relative to fixed cells for individual cells at the resonant frequency of each respective sensor. Qualitatively, this indicates that the live cells are more compliant (softer). To quantitatively illustrate this trend, we fit a normal distribution to our membrane fluctuation data and compare as shown in Fig. 1(b). Live values of membrane fluctuations show a narrower distribution with a mean and standard deviation of 3.86 ± 0.2 nm compared to fixed cells with 2.87 ± 0.1 nm. A paired t-test value (p < 0.001) further confirmed the statistically high significance of the observed fluctuation decrease after fixation. These experimental findings agree well with previous studies on the increase in cell stiffness and viscosity after fixation.^3^ Other cell vibration and membrane fluctuation measurements on red blood cells^24–26^ show significantly higher amplitude values; however, this difference in the amplitude can be attributed to the reasons of integrin-induced tension^27^ in adherent cancer cells.^26^ In addition, resonant sensors in other methods were more compliant (0.01–0.05 N/m) as compared to our resonant sensors (∼19.4 N/m), thus supporting larger deflections of the sensor and the cell membrane.^28^

The amplitude ratio is another key variable in determining the individual cell loss tangent. Figure 1 shows a plot of the observed amplitude ratio (Δ*A*) of live and fixed cells, where there is an increase in the amplitude ratio for the fixed cells over live cells, matching well with previous studies.^23^ A higher amplitude ratio for fixed cells implies that a relative increase in the refractive index (and viscoelasticity) exceeds the relative change in the actual cell-to-sensor amplitude ratio. We can further exemplify the amplitude change for each cell by plotting the live amplitude ratio against the fixed amplitude ratio [Fig. 1(d)] and comparing with a slope of unity (dotted line). Figure 1(e) shows a comparative difference between the loss tangent of live and fixed cells at resonance (∼32.0 kHz and ∼31.8 kHz, respectively), where values above unity indicate a more dissipative, viscous-like state and values below unity indicate an elastic-like state shown graphically in Fig. 1(f). Our observed data show that all cells tend toward a more viscous state with a bias for fixed cells being more viscous than live cells. Fixed cells show an increase in the loss tangent over live cells, indicating that fixed cells exhibit more energy dissipated (viscous) [tan(δ)>1] than energy stored (elastic) within the resonant frequency regime. The average loss tangent values for live and fixed cells are 1.876 ± 0.075 and 4.316 ± 0.126, respectively.

**FIG. 2.**
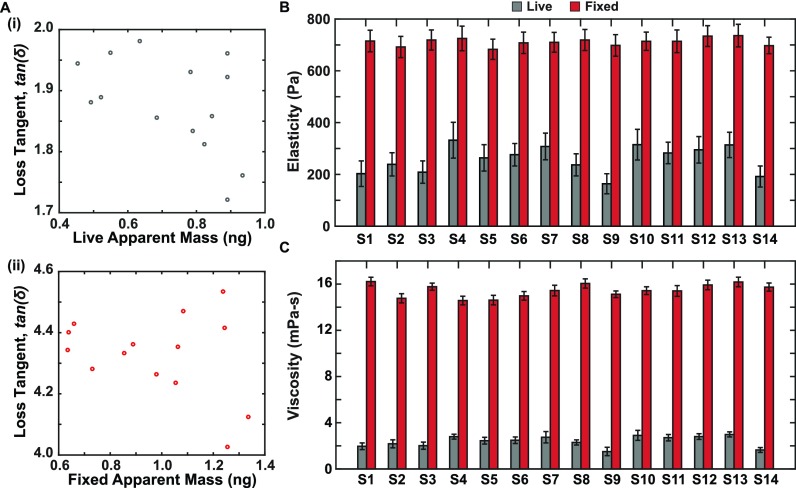
(a) (i–ii) Plots depicting the lack of correlation between the loss tangent $\left[\text{tan}(\delta )=\frac{c_{2}\omega }{k_{2}}\right]$ and the apparent mass of both live and fixed cells further proving that apart from the cell elastic stiffness ${(k}_{2}$) and the viscous effects $\left(c_{2}\right)$ set the dynamics of the system. (b) Elasticity of both live and fixed cells determined independent of mass. (c) Viscosity of both live and fixed cells determined independent of mass.

While a large loss tangent identifies a more viscous (or fluid-like) behavior, it does not automatically mean that the stiffness of the cell is low, rather it means that viscosity dominates over elasticity within our regime of vibration. Our derived tensile storage and loss moduli solution pair ($E^{\prime }=\frac{k_{2}H}{A},\;E^{\prime \prime }=\frac{c_{2}H}{A}\omega$) is used to obtain the apparent elasticity (E) and viscosity (μ) for each cell as shown in Fig. 2, where $E^{\prime }∼\;E$ and $E^{\prime \prime }=\mu \omega \;$(see supplementary material).

Figure 2 compiles the result of our rheological investigation for the 14 different cells. The loss tangent, $\text{tan}\left(\delta \right),$ does not assume a particular cell–sensor geometry. Thus, it is independent of the sensor material property, and the details of contact mechanics provide a quantitative measure of the overall rheological behavior of the sample. Figure 2(a-i-ii) show that there is no correlation between the loss tangent and the apparent mass for (i) live and (ii) fixed cells (R^2^ = 0.232 and 0.083, respectively) to further demonstrate that cell viscosity (*c*_2_) also affects the dynamics of the system, in addition to cell elastic stiffness (*k*_2_). For consistency, we measured the fixed cell mass values (frequency shift) and compared with the same cells before fixation.

The computed apparent elasticity (storage modulus) and viscosity are shown in Figs. 2(b) and 2(c). Live cells showed a lower elasticity of 259 ± 44 Pa, whereas fixed cells showed a higher elasticity of 712 ± 40 Pa. Our reported elasticity range for live cells (164–310 Pa) and higher fixed cell values agree well with other methods.^29–31^ The cell-to-cell variability in viscoelastic moduli elucidates the phenotypic differences in each cell and shows a lack of synchrony in their individual growth cycles despite being the same cell type and probed at the same measurement time. In a previous similar study,^23^ the maximum likelihood of elasticity and viscosity values obtained from an ensemble cell group amplitude and frequency measurements was reported to be 100 Pa and 3.1 mPa s. This work uses an additional optical phase shift measurement (Δϕ) and membrane fluctuation, *A_c_*, of each cell to further derive a more accurate cell-by-cell viscoelasticity solution which is consistent with the order of magnitude in our previous work.^23^ The apparent viscosity values showed a similar trend to elasticity with lower viscosity values for live cells at 2.4 ± 0.3 mPa s and higher viscosity values for fixed cells at 15.4 ± 0.4 mPa s. These viscosity estimates which describe the energy loss comprise the inherent viscosity of cells and surrounding solvent and can be broken apart further for different media conditions. The errors bars in Figs. 2(b) and 2(c) are incurred through optical imaging (area-height ratio estimation), frequency, and loss tangent uncertainty propagation. These findings are consistent with previous studies, showing an increase in cell stiffness and viscosity after fixation.^3,31^

## CONCLUSION

In this study, we derived an expression for the loss tangent measurement of each cell by using a MEMS resonant sensor to capture three observables, frequency shift, amplitude ratio, and phase shift, allowing us to uniquely determine more accurately the viscoelastic moduli and therefore quantify the ratio of energy dissipation upon deformation of each probed cell. The developed model resolves the cell-to-cell heterogeneity in the viscoelastic properties of a sample population, thereby elucidating the phenotypic difference in the growth cycles of individual cells. We found that fixed cells show a substantially larger loss tangent than their live counterparts, revealing that fixed cells are more dissipative and elastic at our resonant frequency. By constraining our governing dynamic equations with an additional optical phase shift and membrane fluctuations of individual cells, we are able to obtain a tighter viscoelastic modulus solution space from our resonant sensors. Our technique can open opportunities to allow for continuous real-time measurements of changes in mass, stiffness, and viscoelasticity over the growth period of single cells, allowing them to serve as a diagnostic tool to identify phenotypes by their mechanical signature and response to mechanical stimulation.

## METHODS

### Measurement scheme: Combining frequency, amplitude, and phase shift

**FIG. 3.**
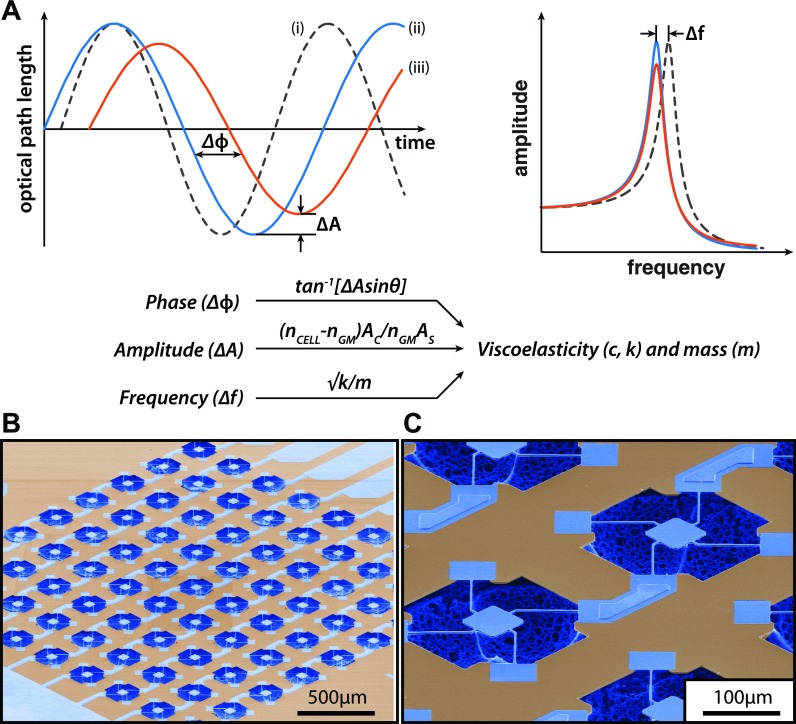
Overview of the measurement scheme. (a) Top left: Plot showing the optical path length, OPL(t), as a function of the apparent phase shift and apparent amplitude increase and time: (i) for a dry-unloaded reference sensor (dotted line), (ii) an outside-cell loaded sensor (blue) (and/or an unloaded wet sensor—for the frequency measurement), and (iii) an inside-cell loaded sensor (red). The phase shift (Δϕ) varies for the loaded sensor outside and inside the cell. Top right: Spectra showing the frequency shift and amplitude change measurement due to dry unloaded and wet unloaded reference (dotted and blue lines) and loaded sensors (red). Bottom: Summary of how the phase shift (Δϕ), amplitude ratio, (ΔA), and frequency shift (Δf) relate to the viscoelastic properties and mass of the cell. (b) SEM Image showing a 9 × 9 array of pedestal sensors. (c) SEM Image showing a close-view layout of individual 60 × 60 μm^2^ sensors.

As shown in Fig. 3(a), the technique to obtain the loss tangent of individual cells involves measuring the frequency (f), amplitude (A), and optical phase shift (Δϕ) in four distinct schemes. First, the response of a dry (unloaded) sensor and an unloaded wet sensor (in-media) is measured to determine the baseline resonant frequency and amplitude. Second, a live cell is loaded on the sensor, and then, the frequency and amplitude shifts are measured, along with optical path length (OPL) variations between laser paths inside and outside the cell. The laser path length will change due to height oscillations of the cell during vibration, resulting in a phase shift.^3,23,32,33^ Figures 3(b)–3(c) show SEM images of our MEMS resonant sensor structure that is electromagnetically actuated to produce vertical motion in the first resonance mode. The details of the resonant sensors and the experimental setup used in this study are described elsewhere.^33^ The viscoelastic effect of HT-29 cells on the resonant frequency and amplitude of the sensor were previously quantified to generate a large space of potential solutions.^23^

### Optical path length (OPL) model

**FIG. 4.**
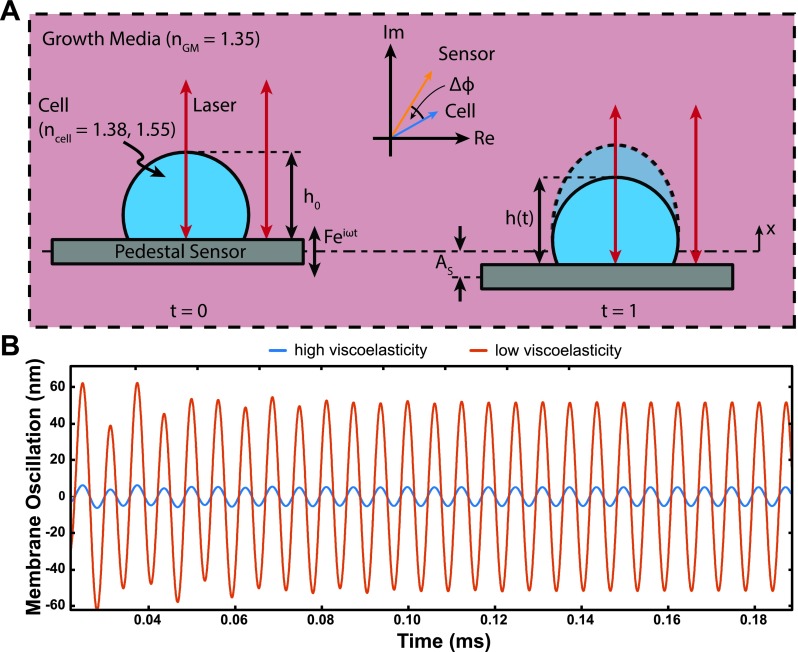
(a) Schematic depicting two time steps of the applied stimuli (${Fe}^{j\omega t}$). Left: Pedestal sensor supporting cell with static height, $h_{0}$ and refractive index, $n_{\text{cell}}.$ Right: the instantaneous cell height oscillation with respect to the sensor, $h(t)=h_{0}+\;A_{C}\;\text{sin}\left(\omega t+\theta_{c}\right);\;A_{C}$ denotes the amplitude of the cell height with respect to the static height, $h_{0}\;$(membrane fluctuation), $\theta_{c}$ denotes the phase difference between cell height oscillation with respect to the applied force, and $A_{s}$ represents the amplitude of the sensor oscillation at resonance frequency, ω. The shift (Δϕ) indicates the observed optical phase shift between two light paths, one through the cell and the other directly on the sensor (red lines with arrows). (b) Simulation of both transient and steady state membrane fluctuations of a cell with high and low viscoelasticities, i.e., the ratio, $\frac{c_{2}}{k_{2}}\;,$ is held constant, while the viscoelastic coefficients ($c_{2}\;k_{2}$) are scaled for high and low viscoelasticities.

The current study takes a step further by focusing on combining the complementary optical path phase-shift and membrane fluctuation measurements to improve the stiffness and viscous dissipation solution space. As was previously established, a laser Doppler vibrometer (LDV) can measure a time-derivative of the OPL and therefore can determine the membrane oscillation of the cell.^21,34^ Figure 4(a) shows a schematic overview of the resonant measurement method and represented variables in the following equations:

$$\begin{array}{c} OPL\left(t\right)=\sum n_{i}d_{i}\left(t\right)\;{n_{GM}}^{*}\;\left(P_{\text{sensor}\;}-h\left(t\right)\right)+n_{\text{cell}}\;*h(t)\\ \approx \;n_{GM}{A_{s}}^{*}{\left(1+\Delta A\right)}^{*}\text{sin}\;\left(\omega t+\Delta \phi \right)+\text{const}. \end{array},$$ (1)

$$\Delta A=\;[\frac{{(n}_{\text{cell}\;-\;}n_{GM})\;}{n_{GM}}\;*\frac{A_{c}}{A_{s}}],$$ (2)

$$\Delta \phi =\text{arctan}[\frac{{(n}_{\text{cell}\;-\;}n_{GM})\;}{n_{GM}}\;*\frac{A_{c}}{A_{s}}*\;\text{sin}(\theta )],$$ (3)

where *n_cell_* represents the refractive index of the cell, *n_GM_* represents the refractive index of the media, *A_c_* represents the amplitude of the cell height oscillation with respect to the cell initial height also denoted as the cell membrane fluctuation (amplitude), *A_s_* represents the amplitude of the sensor oscillation, θ represents the phase of the cell height oscillation with respect to the sensor, ω represents the oscillating resonant frequency, and Δϕ is our measured maximum phase shift of the OPL. $P_{\text{sensor}\;}\left(t\right)$ and $h\left(t\right)$ are the instantaneous sensor position and cell height.

### Membrane fluctuation determination: Analytical estimation and simulation

We can uniquely compute the individual cell membrane fluctuation at resonance, *A_c_* for one cell per sensor, which overcomes the initial bulk estimate limitation. This is calculated via Eq. (2) by estimating *A_s_* = 180 pm (from measured velocity and oscillating frequency) for a 35 nN (1 Vrms) excitatory input in media, *n_GM_ = *1.35 and *n_cell_* = 1.38 and 1.55 for live and fixed cells, respectively.^21^ To better understand how the changes in cell membrane fluctuations occur, we modeled our sensor-cell system as a two-degree-of-freedom (2-DOF) suspended mass model where the cell mass (*m*_2_) is considered a Kelvin-Voigt viscoelastic solid with elastic stiffness (*k*_2_) and the viscous coefficient (*c*_2_) connected to the sensor and the sensor mass (*m*_1_) is connected to the fixed substrate by a second Kelvin-Voigt spring-damper (*k*_1_, *c*_1_) (see supplementary material). The model assumes an oscillatory force *F*(*t*) applied to the sensor mass. Figure 4(b) illustrates the effect of both high and low viscoelastic cell mass (*m*_2_) loaded sensors on the membrane fluctuations by simulating the time-dependent transient and steady state dynamic responses at a representative resonant frequency (ω) of our sensors. In these simulations, the ratio $\frac{\;c_{2}}{\;k_{2}}\;$ is held constant and the stiffness/dissipation pair (*k*_2_*, c*_2_) is scaled for cases of high and low viscoelasticities. These simulations depend on a few required assumptions that the cell membrane is homogeneous and smooth as well as the average cell-surface oscillation is calculated in the steady state.

### Data analysis and cell culture

We combined interferometric techniques to measure the viscoelasticity of both live and fixed individual HT-29 cells. Cells were cultured and fixed on resonant sensors as described in previous work^33^ (see supplementary material). To fully investigate the mechanical response of live cells compared to fixed cells, we started with the data collection of the necessary established methods, including amplitude ratios,^23^ phase shift,^21^ and frequency shift,^33^ to ultimately convey a meaningful rheological translation. Ethics approval is not required for this study.

We model the 2-DOF system behavior as described fully in the supplementary material. The experimental observables based on the LDV measurements are related to mechanical quantities as given in Eqs. (2)–(3). Knowing the mechanical amplitude ratio and the phase shift between the cell and sensor oscillations and calibrating sensor mass, stiffness, and dissipation allow us to obtain cell viscoelastic properties at the probed resonant frequencies.

### Estimation of viscoelastic coefficients (*k*_2_, *c*_2_)

The mechanical phase difference (θ) is related to cell viscoelastic properties that we extract by considering the full 2-DOF suspended mass system. To estimate the viscoelastic coefficients (*k*_2_, *c*_2_), our microresonator is modeled as a 2-DOF Kevin-Voigt model as described in the following equation in simplified matrix form:

[(k1+k2−m1ω2)+(c1+c2)ωj−k2−c2ωj−k2−c2ωj(k2−m2ω2)+c2ωj]{AsAc} ejωt= {F0} ejωt. (4)

Note that the physical quantities of interest are found by taking the imaginary components, e.g., $F\left(t\right)=F\text{sin}\;\omega t=I_{m}\left\{Fe^{j\omega t}\right\}$, where $A_{s}={A_{s}^{R}}_{\;}+{{jA}_{s}^{I}}_{\;}$ and $A_{c}={A_{s}^{R}}_{\;}+{{jA}_{c}^{I}}_{\;}$ are the vector amplitudes of the sensor and cell states $x_{\text{sensor}}$ and $x_{\text{cell}},$ respectively, and ${Fe}^{j\omega t}$ is the input force. Equation (4) is further decomposed as the instantaneous sensor and cell responses in the following equations:

$$x_{\text{sensor}}\left(t\right)=\;\left|A_{s}\right|\;\text{sin}\left(\omega t\;+\;\theta_{s}\right),$$ (5a)

$$x_{\text{cell}}\left(t\right)=\left|A_{c}\right|\;\text{sin}\left(\omega t\;+\;\theta_{c}\right),$$ (5b)

where $\theta_{s}={\text{tan}}^{-1}\left\{\frac{{(A}_{s}^{I})}{{(A}_{s}^{R})}\right\}$ and $\theta_{c}={\text{tan}}^{-1}\left\{\frac{{(A}_{c}^{I})}{{(A}_{c}^{R})}\right\}$. Here, $\theta_{s}$ and $\theta_{c}$ denote the phase differences between the sensor and cell height oscillation with respect to the excitatory force, $F\left(t\right)=F\text{sin}\;\omega t$. Our modeled cell-sensor phase difference

$$\theta =\;\theta_{c}-\;\theta_{s},$$ (6)

and modeled cell-sensor amplitude ratio become

$$\Delta A=\frac{{(n}_{\text{cell}\;-\;}n_{GM})\;}{n_{GM}}\;\frac{\left|A_{c}\right|}{\left|A_{s}\right|}.$$ (7)

The observed amplitude ratio (Δ*A*) and mechanical phase shift (θ) inferred from Eq. (2) are substituted to simultaneously solve Eqs. (6) and (7) for *k*_2_ and *c*_2_ (see supplementary material).

### Loss tangent estimation: Using area-to-height

The loss tangent, $\text{tan}\left(\delta \right),$^35,36^ is a dimensionless parameter that measures the ratio of energy dissipated to energy stored and can be used as a viscoelasticity indicator of our probed cell. It is totally independent of the mass of each cell and is different from our estimated cell-sensor mechanical phase shift $\left(\theta \right),$ which largely depends on our three observables, frequency (f), amplitude ratio (ΔA), and optical phase shift (Δϕ). Furthermore, the loss tangent, $\text{tan}\left(\delta \right)=\frac{c_{2}\omega }{k_{2}}$, does not assume any cell geometry. However, the viscoelastic coefficients ($k_{2},\;c_{2})$ can be related to the apparent inherent viscoelastic moduli (*E, μ*) by $k_{2}=\frac{EA}{H}$ and $c_{2}=\frac{\mu A}{H}$. The ratio $\frac{A}{H}$ denotes the area-to-height information of each cell, where the average cell area is 250 μm^2^ with an estimated cell height of 8 μm (see supplementary material).^33^

## SUPPLEMENTARY MATERIAL

See supplementary material for further details of the mechanical model dynamics used in estimating the viscoelastic coefficients (*k*_2_, *c_2_*), each cell mass calibration protocol, colon cancer cell (HT-29) culture protocol, and viscoelastic modulus error characterization. Also, additional data on our observed amplitude ratio, phase shift including characterization of excitation against the shift in the optical path length, and cell-sensor amplitudes are presented.
